# On the Impact of Residual Strains in the Stress Analysis of Patient-Specific Atherosclerotic Carotid Vessels: Predictions Based on the Homogenous Stress Hypothesis

**DOI:** 10.1007/s10439-024-03458-4

**Published:** 2024-02-13

**Authors:** Alessandro Mastrofini, Michele Marino, Eva Karlöf, Ulf Hedin, T. Christian Gasser

**Affiliations:** 1 Department of Civil Engineering and Computer Science Engineering, University of Rome Tor Vergata, Rome, Italy; 2 Vascular Surgery, Department of Molecular Medicine and Surgery, Karolinska Institute, Stockholm, Sweden; 3 KTH Solid Mechanics, School of Engineering Sciences, KTH Royal Institute of Technology, Stockholm, Sweden; 4 Faculty of Health Sciences, University of Southern Denmark, Odense, Denmark

**Keywords:** Tissue stress, Computational biomechanics, Atherosclerotic disease, Plaque rupture risk, Growth & remodeling

## Abstract

The identification of carotid atherosclerotic lesion at risk for plaque rupture, eventually resulting in cerebral embolism and stroke, is of paramount clinical importance. High stress in the fibrous plaque cap has been proposed as risk factor. However, among others, residual strains influence said stress predictions, but quantitative and qualitative implications of residual strains in this context are not well explored. We therefore propose a multiplicative kinematics-based Growth and Remodeling (G&R) framework to predict residual strains from homogenizing tissue stress and then investigate its implication on plaque stress. Carotid vessel morphology of four patients was reconstructed from clinical Computed Tomography-Angiography (CT-A) images and equipped with heterogeneous tissue constitutive properties assigned through a histology-based artificial intelligence image segmentation tool. As compared to a purely elastic analysis and depending on patient-specific morphology and tissue distributions, the incorporation of residual strains reduced the maximum wall stress by up to $30\%$ and resulted in a fundamentally different distribution of stress across the atherosclerotic wall. Regardless residual strains homogenized tissue stresses, the fibrous plaque cap may persistently be exposed to spots of high stress. In conclusion, the incorporation of residual strains in biomechanical studies of atherosclerotic carotids may be important for a reliable assessment of fibrous plaque cap stress.

## Introduction

Atherosclerosis, the most common cardiovascular disease [1], poses a significant burden on global healthcare systems. Causing strokes, heart attacks, and many other serious cardiovascular events, atherosclerosis characterizes the leading cause of death and disability worldwide [2].

While other mechanisms have also been proposed [3], atherosclerosis is commonly believed to be an inflammatory tissue response to endothelial cell dysfunction, processes that are also strongly influenced by mechanical factors, such as blood pressure and wall shear stress [4, 5]. Atherosclerotic lesions present at very diverse morphologies, thereby demanding patient-individual risk assessment [6]. However, no diagnostic method, or combination of methods, can accurately determine whether an asymptomatic atherosclerotic lesion is vulnerable with risk for plaque rupture, cerebral embolism, and stroke. It explains why the stroke preventive effect of surgery (Carotid EndArterectomy; CEA) in patients with asymptomatic carotid stenosis is modest (1/20–1/30), and even in symptomatic patients, six to eight CEA interventions are needed to prevent a single stroke. Given the lack of information for individual decision-making, present guidelines [7] are derived from evidence at the group level, resulting in poor accuracy to identify patients who would gain from clinical treatment. In conclusion, currently implemented patient treatments are based on low to moderate grades of evidence and therefore continuously questioned [8–10].

Computational biomechanics, mainly based on Finite Element Method (FEM) studies, has emerged as a powerful tool to explore atherosclerotic carotid disease, enabling the prediction of tissue mechanical stresses toward the study of plaque formation, enlargement, and rupture [11, 12]. Such models critically depend on the mechanical description of normal and pathological vascular tissues, a choice of direct implication on stress predictions [13, 14]. Regardless of advances made in the acquisition of patient-specific tissue morphology [15], tissue-specific biomechanical properties of patient-specific lesions remain unknown.

For a long time, opening angle experiments illustrated the existence of residual strains in vascular tissue [16], and more recently Growth and Remodeling (G&R) studies uncovered mechanisms behind the development of residual strains [17, 18]. Regardless residual strains are known to influence tissue stress computations [19, 20], they are commonly not considered in biomechanical models of atherosclerotic blood vessels. Residual strains are multidimensional, cannot be measured directly, and no consensus has been emerged of how to include residual strains in patient-specific biomechanical blood vessel models [19, 20].

There is clear evidence that the average tension in the normal vessel wall remains reasonably constant across the individual life span and different biological species [21], information that led to the homogenous stress hypothesis [22, 23]. According to it, residual stresses can be seen as the result of vascular tissue G&R activities in an attempt to reduce stress gradients. The homogenous stress hypothesis has therefore been considered together with classical physical governing laws, such as mechanical equilibrium, to account for local residual strain fields in vascular tissue stress predictions [18, 24]. We further elaborate along this concept and propose a G&R computational framework with application to stress analysis of patient-specific atherosclerotic carotid blood vessels.

## Methods

### Kinematic Description and Elastic Response

We introduce an (incompatible) intermediate configuration $\Omega _0$, in between the vascular tissue’s reference configuration $\tilde{\Omega }_0$ and its current configuration $\Omega$. Following an exact kinematics description, the deformation gradient then reads ${\textbf{F}}={\textbf{F}}_{\textrm{e}}{\textbf{G}}$, where the growth tensor ${\textbf{G}}$ represents the growth-related deformation from ${\tilde{\Omega }}_0$ to $\Omega _0$, whereas ${\textbf{F}}_{\textrm{e}}$ describes non-growth-related deformation between ${\Omega }_0$ and $\Omega$ [5, 25]. The non-growth-related deformation is assumed to be purely elastic and modeled by the incompressible isotropic Yeoh strain energy density function [26]

1 $$\begin{aligned} \Psi =\sum _{i=1}^3 c_i\left( \bar{I}_{1 \textrm{e}}-3\right) ^i, \end{aligned}$$

where $c_1$, $c_2$ , and $c_3$ are material parameters (specific for each tissue component), and $\bar{I}_{1\textrm{e}}={\text {Tr}}\left( {\bar{\textbf{C}}}_{\textrm{e}} \right)$ denotes the first invariant of the isochoric elastic right Cauchy-Green deformation tensor $\bar{{\textbf{C}}}_{\textrm{e}}=J_e^{-2/3}{\textbf{F}}_{\textrm{e}}^T{\textbf{F}}_{\textrm{e}}$. As previously mentioned, the incompressibility constraint $J_{\textrm{e}}=\textrm{det}(\textbf{F}_{\textrm{e}})=1$ is enforced on the elastic part of the deformation.

**Table 1:** Material parameters for Yeoh’s constitutive model (1) in the description of MATriX tissue (MATX), CALCification (CALC), Lipid-Rich Necrotic Core (LRNC), and Intra-Plaque Hemorrhage (IPH) of atherosclerotic carotid blood vessels

	$c_1$ [kPa]	$c_2$ [kPa]	$c_3$ [kPa]
MATX	23.5	126	112
CALC	302.1	$-$228	261
LRNC/IPH	29.6	$-$33.2	128.5

Parameters are taken from the literature [27]

### Image Reconstruction and FEM Model Generation

**Fig. 1:**
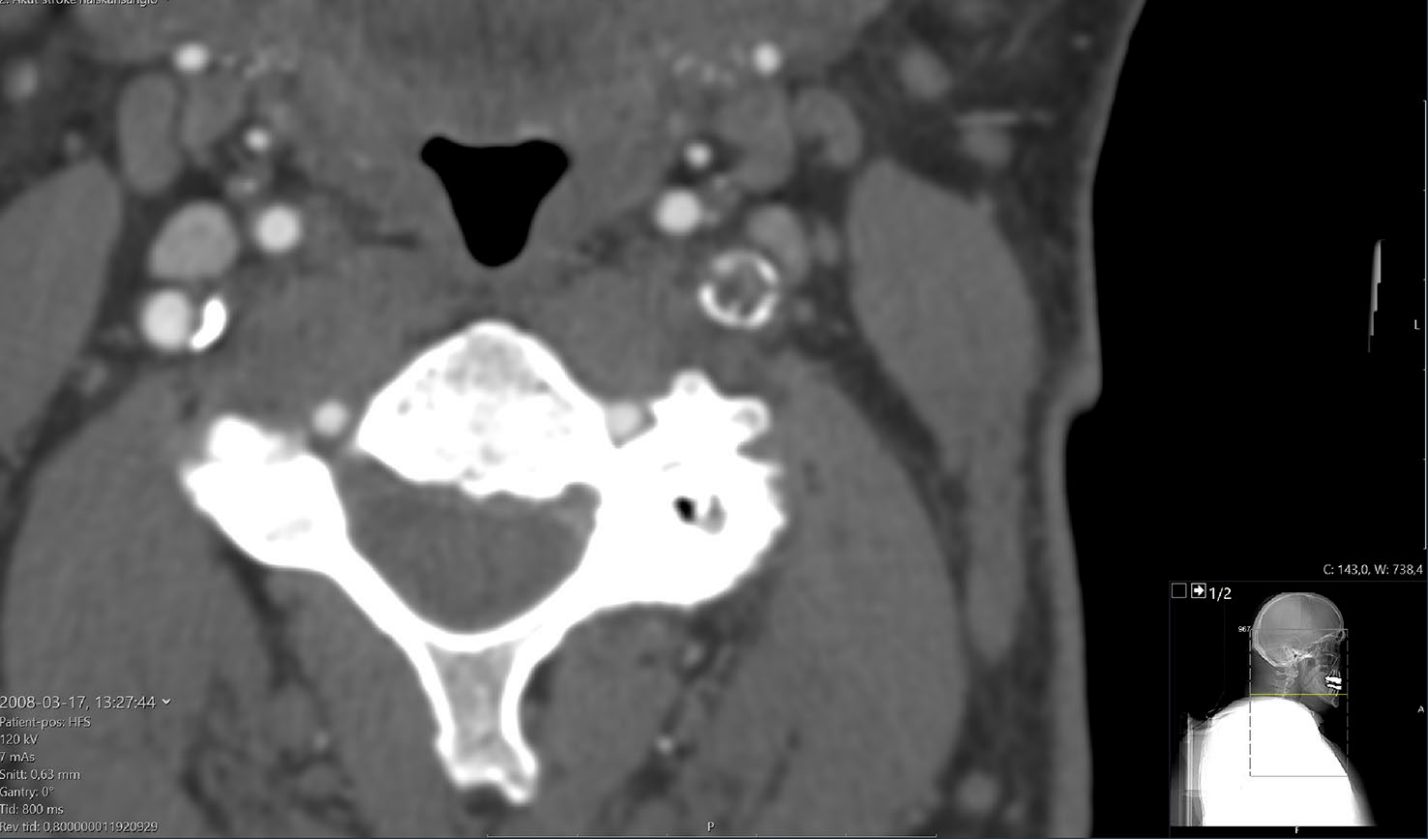
Selective computed tomography-angiography (CT-A) image slice acquired through the clinical examination of patient 1. The location of the image slice is indicted by the inset at the bottom right, and patient characteristics are listed in Table 3

Being part of the routine clinical examination, Computed Tomography-Angiography (CT-A) images of the patient’s neck were taken at an in-plane resolution of $0.5\times 0.5$ mm^2^ and a slice thickness of 0.63 mm, see Fig. 1. Carotid arteries were then segmented from said CT-A images toward the generation of individual plaque morphologies. We used ElucidVivo [28], a software utilizing histology-based artificial intelligence algorithms to discriminate between Lipid-Rich Necrotic Core (LRNC), Intra-Plaque Hemorrhage (IPH), CALCifications (CALC), and the surrounding MATriX tissue (MATX). This information was then converted into a 3D mesh that allows for structural FEM analysis of the blood vessel. We used the iso2mesh [29] package in Matlab [30], in combination with postprocessing operations to avoid undesired holes in the vessel’s wall.

The mesh was then imported into COMSOL Multiphysics [31] for FEM calculation. As the different tissues components were not modeled by distinct FEM bodies/domains, the entire vessel wall was meshed at first and the material parameters of the individual tissues then prescribed over said discretized domain. Specifically, each FEM mesh node was assigned material parameters as reported in Table 1, which were then interpolated within the individual finite element. Hence, the sharp interface in material properties between different tissue components has been replaced by a continuous material transition within a single finite element.

Blood pressure *p* was applied as a follower load (Neuman boundary condition) on the luminal surface, and Dirichlet boundary conditions delineated the proximal and distal domain boundary, respectively. The FEM mesh consisted of tetrahedral mixed elements with quadratic interpolation for displacements and linear interpolation for the Lagrange multiplier that enforced the incompressibility constraint on the elastic part of the deformation. A mesh sensitivity analysis concluded that our models required approximately 200k finite elements.

### Implementation of Tissue Growth and Remodeling

G&R relies on the biological tissue’s proper metabolic functioning. The remodeling algorithm was therefore only applied to vascular matrix tissue (MATX), while all the other tissue components (CALC, LRNC/IPH) have been represented by inert materials, that is assuming ${\textbf{G}}={\textbf{I}}$. On the other hand, following the homogeneous stress hypothesis [22, 32], the growth tensor ${\textbf{G}}$ in MATX tissue evolves toward minimizing the gradient of the first principal Cauchy stress $\sigma _1$ within the tissue [24]. As the time scale of growth is much larger than the cardiac cycle, said stress refers to the vessel at Mean Arterial Pressure (MAP).

Assuming a null rigid rotation during growth (rigid rotation is accommodated by ${\textbf{F}}_{\textrm{e}}$), the growth tensor is computed according to an updated Lagrangian framework where the incremental growth is defined as:

2 $$\begin{aligned} \Delta {\textbf{G}}=\, & {} {\textbf{I}}+\alpha \underbrace{ \left( \xi _1 {\textbf{e}}_1 \otimes {\textbf{e}}_1+ \xi _2 {\textbf{e}}_2 \otimes {\textbf{e}}_2 + \xi _3 {\textbf{e}}_3 \otimes {\textbf{e}}_3\right) }_{\varvec{\Lambda }} \;\;\;\text{ with }\;\;\;\nonumber \\ \xi _i=\, & {} {\textbf{n}}_1\cdot {\textbf{e}}_i. \end{aligned}$$

It describes anisotropic growth, preferentially along the first principal direction ${\textbf{n}}_1$ of the Cauchy stress tensor at the last equilibrated configuration.

In fact, the two-point second-order tensor $\varvec{\Lambda }$ aligns growth with the first principal stress direction in the global reference system $({\textbf{e}}_1,\,{\textbf{e}}_2,\,{\textbf{e}}_3)$, and $\xi _i$ therefore represents the projection of ${\textbf{n}}_1$ into the base vectors ${\textbf{e}}_i; i=1,2,3.$ Unrelated to the growth direction, $\alpha$ represents a growth factor, defined as:

3 $$\begin{aligned} \alpha =\frac{1}{c} \frac{{\sigma }_1-\bar{{\sigma }_1}}{\max \left[ {\sigma }_1,\bar{{\sigma }_1}\right] }, \end{aligned}$$

where $\sigma _1$ is the first principal stress and *c* is a regularization parameter. Moreover, $\bar{{\sigma }}_1= (\sum _{i=1}^N \sigma _1^i)/N$ denotes the average of the first principal Cauchy stress in the MATX tissue, where *N* denotes the number of the corresponding finite element nodes.

**Table 2:** Nested iteration at each pressure increment $\Delta p$ up to the Mean Arterial Pressure (MAP) toward the computation of the incremental growth tensor $\Delta {\textbf{G}}$

1. Solve the global mechanical problem, i.e., ensure equilibrium, and update mesh	
2. Compute the first principal Cauchy stress $\sigma _1^i$ and stress direction ${\textbf{n}}_1^i$ at the $i=1,\ldots ,N$ finite element nodes of the MATX tissue	
3. Compute the average Cauchy stress in MATX tissue $\bar{{\sigma }}_1= (\sum _{i=1}^N \sigma _1^i)/N$	
4. If $(\sum _{i=1}^N \vert \sigma _1^i - {\bar{\sigma }}_{1}^i\vert )/(N {\bar{\sigma }}_{1}^i)> tol$ and $n_{\textrm{iter}} < max_{\textrm{iter}}$:	
4.1 Compute the growth factor	
$\alpha ^i=\frac{1}{c} ({\sigma }_1^i-\bar{{\sigma }_1})/\left(\max \left[ {\sigma }_1^i,\bar{{\sigma }_1}\right] \right)$ at the *i*-th finite element node	
4.2 Compute the incremental growth tensor	
$\Delta {\textbf{G}}^i\leftarrow {\textbf{I}} + \alpha ^i(\xi _1^i {\textbf{e}}_1 \otimes {\textbf{e}}_1 + \xi _2^i {\textbf{e}}_2 \otimes {\textbf{e}}_2 + \xi _3^i {\textbf{e}}_3 \otimes {\textbf{e}}_3)$ with	
$\xi _j^i={\textbf{n}}_1^i\cdot {\textbf{e}}_j$ at the *i*-th finite element node	
4.3 Go to 1.	
Else:	
Go to 1 with $p\rightarrow p+\Delta p$	

The algorithm implements the homogenous stress hypothesis through an updated Lagrangian formulation

For each increment of external loading, and thus pressure increment $\Delta p$ up to MAP, a nested iteration is used to compute the growth tensor increment $\Delta {\textbf{G}}$, see Table 2. In general, global equilibrium is incompatible with a homogenous stress state. The stress increment $\vert \sigma _1 - {\bar{\sigma }}_{1}\vert$ will therefore not always approach zero, and the iteration is terminated when the number of iterations exceeds the limit of $max_{\textrm{iter}}$. In this case, our iteration may be seen as an optimization problem; the stress state achieved is as homogenous as ‘permitted’ by the equilibrium. Given the highly non-linear character of the structural problem, the substitution of the regularization term 1/*c* at Step 4.1 in Table 2 by *f*/*c*, where the function *f*(*p*) increases quadratically from 0 (at $p=0$) to 1 (at $p=$MAP), improves the numerical robustness, i.e., allows for larger pressure steps $\Delta p$.

## Results

At first, and primarily toward the exploration of the numerical performance of our algorithm, we studied simple, hypothetical vessel geometries described in Sect. 3.1. Results from patient-specific case studies are then presented in Sect. 3.2. For all numerical studies, the internal pressure *p* has been linearly ramped up to MAP $=75$ mmHg with increments $\Delta p=9.375$ mmHg. As long as $p<$ MAP, only one iteration was used to update the growth tensor ${\textbf{G}}$ (i.e., $max_{\textrm{iter}}=1$), while at $p=$ MAP, $max_{\textrm{iter}}=6$ and $tol<0.5\%$ determined the iterative identification of ${\textbf{G}}$. The range $c \in [2,10]$ of the regularization parameter resulted in an overall good (stable) numerical performance, although this parameter is to some extent problem-dependent.

### Hypothetical Vessel Geometries

**Fig. 2:**
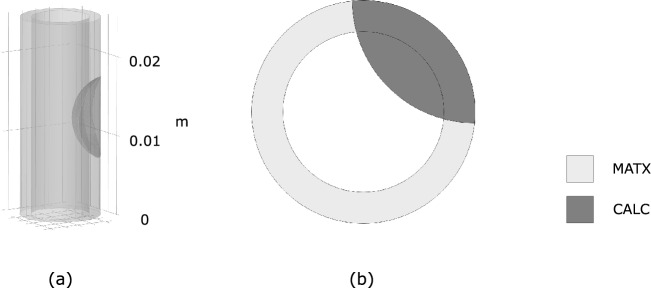
Hypothetical carotid blood vessel presenting MATriX tissue (MATX) and CALCification (CALC)

The investigated hypothetical vessel geometries displayed dimensions similar to human carotid vessels. In addition to an ideal cylindrical geometry made of MATX properties, we also considered a cylindrical vessel that contained a portion of CALC tissue, while all remaining wall tissue was again assigned homogeneous MATX properties, see Fig. 2.

**Fig. 3:**
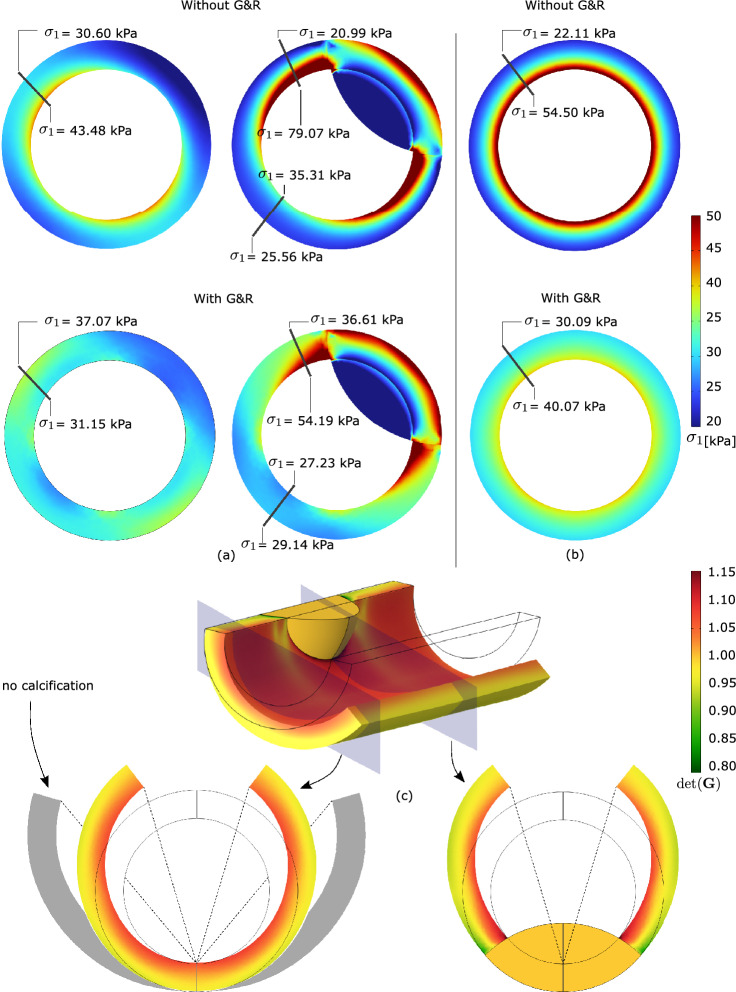
Implications of residual strains on wall stress in a cylindrical vessel segment without and with CALCification (CALC). Model geometries are defined in Fig. 2 and tissue Growth and Remodeling (G&R) is limited to MATriX (MATX) tissue. The inset marks the analyzed cross-sections of the model containing CALC. Top: First principal Cauchy stress $\sigma _1$ at Mean Arterial Pressure (MAP) of 75 mmHg without and with the consideration of Growth and Remodeling (G&R), and thus with and without the evolution of residual strains. The stress pattern appears similar across both models; residual strains reduced the peak stresses as well as the stress gradient across the wall. Bottom: Opening angles illustrating the existence of residual strains. Given the model with CALC, the determinant of the growth tensor $\textrm{det}({\textbf{G}})$ denotes local tissue growth. The presence of CALC strongly reduces the opening angle as compared to the non-calcified model, shown in gray

As compared to the analysis without an evolution of residual stresses, our G&R algorithm predicted a more homogenous stress distribution across the vessel wall, see Fig. 3(top). With reference to the model containing CALC tissue, remarkable changes in the stress distribution at the interface between MATX and CALC are also observed, highlighting the impact of material heterogeneities in stress calculations.

In addition to the wall stress analysis at MAP, the implications of residual strains were studied in terms of the resulting opening angle, see Fig. 3(bottom). As reported elsewhere [24], the vessel was unloaded from MAP to zero blood pressure and then allowed to open-up toward releasing residual stresses. The ideal cylindrical vessel showed an opening angle of 79^∘^, a value much larger than the angle of 33^∘^ predicted by the model that contained a portion of CALC tissue. Interestingly, the opening angle of the model with CALC remains relatively constant along the vessel, and the calcified tissue therefore had a strong non-local effect on the opening angle.

### Patient-Specific Carotid Bifurcations

Four patient-specific atherosclerotic carotids were analyzed with the patient characteristic listed in Table 3.

**Table 3:** Characteristcs of the patient-specific atherosclerotic carotid arteries

Patient	Age	Sex	Symptoms	Smoking	Co-morbidites
1	54	M	AS	No	Hypertension, angina, lipid-lowering therapy
2	76	F	TIA	Yes	Hypertension
3	73	M	AF	No	Hypertension, earlier TIA, lipid-lowering therapy
4	69	M	AS	No	Hypertension, angina, earlier TIA, lipid-lowering therapy

*AS* Asymptomatic stenosis; *TIA* Transient Ischemic attack, *AF* Amaurosis fugax

#### Patient 1

**Fig. 4:**
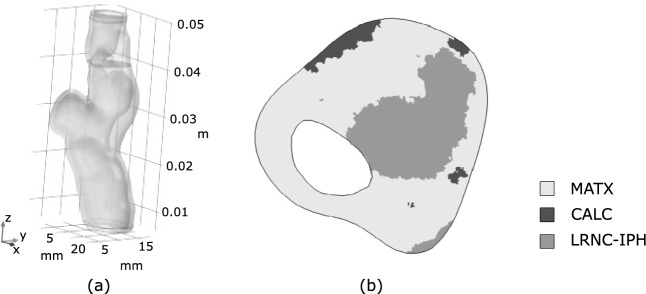
Patient 1: **a** 3D reconstruction of the vessel from Computed Tomography-Angiography (CT-A); **b** Morphology at the cross-section of maximum stenosis with different tissues highlighted at different gray levels

The carotid of the first patient is shown in Fig. 4, and a morphological analysis revealed that MATX, CALC and LRNC/IPH covered 70%, 17%, and 13% of the reconstructed volume, respectively. At the cross-section of maximum stenosis, we are looking at a considerably narrowed lumen and a vessel wall containing a massive portion of lipid-rich tissue (LRNC/IPH).

**Fig. 5:**
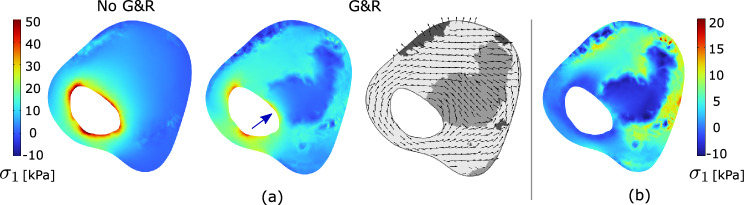
Implications of residual strains on the first principal Cauchy stress $\sigma _1$ in the carotid vessel of Patient 1. Data are analyzed at the cross-section of maximum stenosis and tissue Growth and Remodeling (G&R) is limited to MATriX (MATX) tissue. **a** Stress predictions at Mean Arterial Pressure (MAP) without and with the consideration of G&R. The direction of the first principal stress is also illustrated. The inclusion of residual strains reduced peak stresses by approximately 30%, resulting in a more homogenized stress distribution within the tissue. While the soft region formed by the Lipid-Rich Necrotic Core (LRNC) and the Intra-Plaque Hemorrhage (IPH) redistributes the stress, a stress localization in the plaque cap remains, as indicated by the blue arrow. **b** Residual Cauchy stress at the zero-pressure configuration, and thus following unloading to zero blood pressure of the configuration that developed at MAP according to G&R

**Fig. 6:**
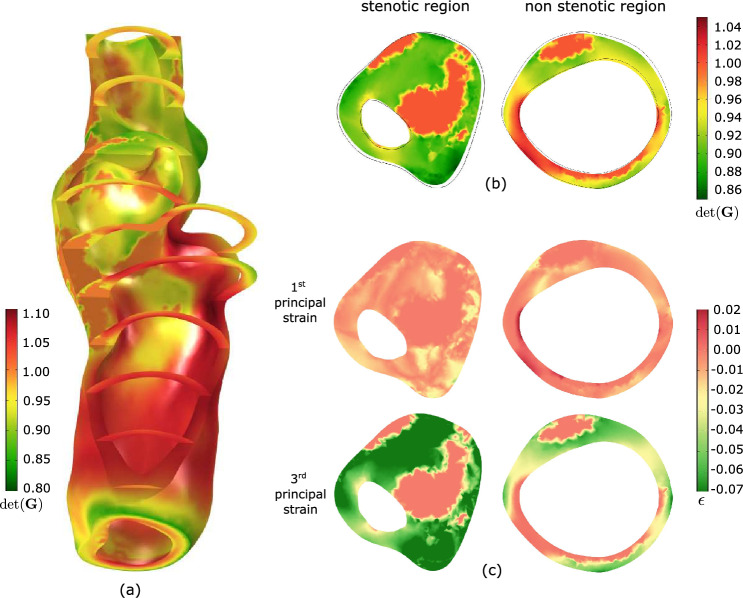
Growth and Remodeling-related (G&R-related) deformations within the plaque tissue of Patient 1. Configurations are shown at zero blood pressure. **a**, **b** Determinant of the growth tensor $\textrm{det}({\textbf{G}})$ indicating G&R-related local volume change over the vessel segment and at selective cross-sections, respectively. The black edges in (**b**) represent the initial reference configuration. **c** First and third principal residual strain at selective cross-sections

**Fig. 7:**
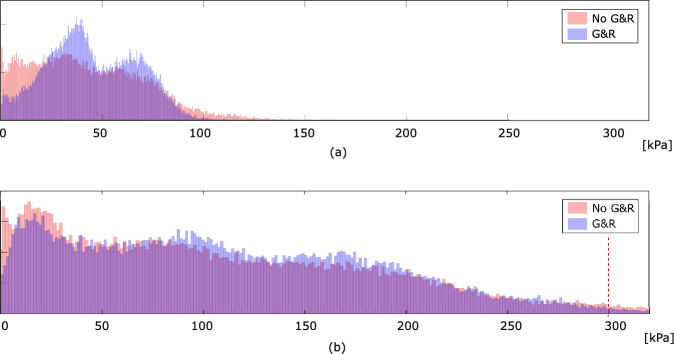
Distribution of the first principal Cauchy stress in the vessel wall of Patient 1. Data refer to loading at the Mean Arterial Pressure (MAP) of 75 mmHg (**a**) and the systolic pressure of 180 mmHg (**b**), respectively

The predictions of the first principal stress at MAP of 75 mmHg, with and without the consideration of residual stresses (with and without G&R), differ remarkably, see Fig. 5a, b. The inclusion of residual strains results in a more homogenous stress distribution and shows the ability to reduce the peak stress by approximately 30% in the luminal tissue. Remarkably, a spot of high stress remains in the fibrous cap. Despite not being explicitly modeled, we refer to fibrous cap as the morphological part of the plaque close to the luminal border.

Figure 7a confirms these observations; the first principal stress concentrates around the average stress, defining a more homogenized stress distribution. In addition, the inclusion of residual strains shifts the stress from the lipid-rich tissue toward the matrix tissue, LRNC/IPH toward MATX, see Fig. 5a.

Figure 6 displays G&R-related deformations within the plaque tissue. Here, Fig. 6a, b illustrates the determinant of the growth tensor $\textrm{det}({\textbf{G}})$, whereas Fig. 6c reports the first and third principal residual strains, respectively. Residual strains are multidimensional and highly hetereogenously distributed over the plaque tissue. At the stenotic site, residual strains are generally negative and the arterial cross-section shrinks with respect to the initial configuration. Away from the stenosis and approaching tube-like vessel segments, the characteristic distribution of residual strains is observed, negative outside and positive inside. Here, an almost zero net change of tissue volume across the wall is maintained.

As it is the highest stress during the cardiac cycle that eventually leads to plaque rupture, wall stress at the systolic blood pressure of 180 mmHg was also investigated. Given this task, the MAP-based residual stress state has either been included or been excluded, and the stress predictions then compared among both assumptions. Similar to the aforementioned stress predictions entirely at MAP, the inclusion of residual stress decreases the peak stress also at systolic blood pressure, see Fig. 7b. Both models predicted stress beyond 300 kPa, a value the literature generally associates with the risk of plaque rupture [27]. However, the inclusion of residual stress reduced the tissue volume that is exposed to stress beyond said value from 4.3% to, respectively, 2.2%, see Fig. 7b.

**Fig. 8:**
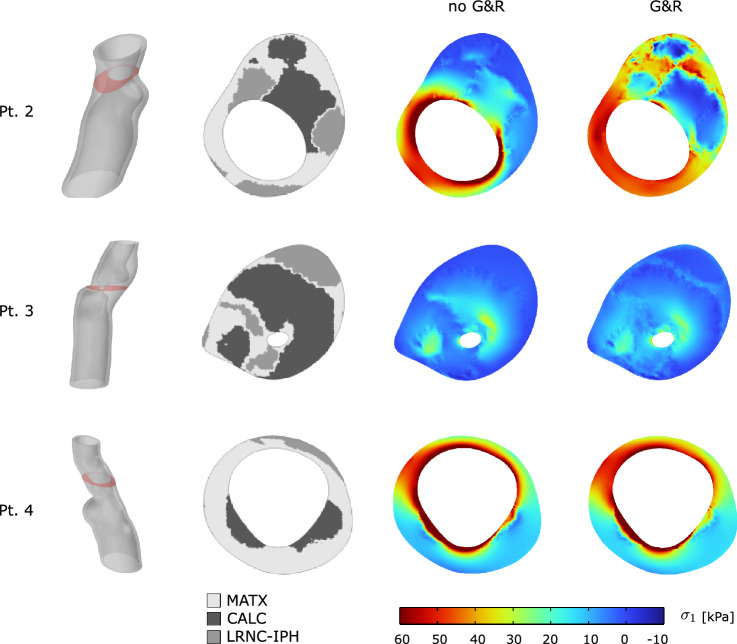
Implication of residual strains on carotid wall stress predictions with moderate (Patient 2), severe (Patient 3), and mild (Patient 4) stenosis. Column 1: 3D reconstruction from Computed Tomography-Angiography (CT-A); Column 2: Morphology at the cross-section of maximum stenosis with different tissues highlighted at different gray levels; Column 2 and 3: First principal Cauchy stress $\sigma _1$ at the Mean Arterial Pressure (MAP) of 75 mmHg without and with the evolution of residual strains within MATriX tissue (MATX)

**Fig. 9:**
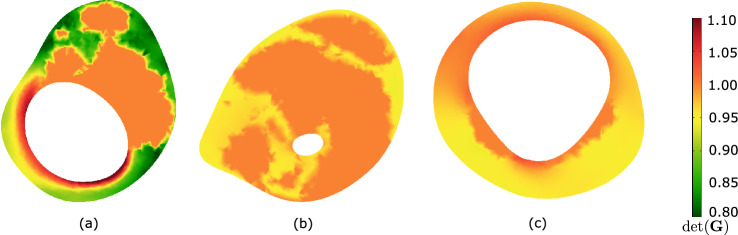
Determinant of the growth tensor $\textrm{det}({\textbf{G}})$ indicating Growth and Remodeling-related (G&R-related) local volume change at the cross-section of maximum stenosis. **a** Patient 2, **b** Patient 3, **c** Patient 4. See Fig. 6b for Patient 1

**Fig. 10:**
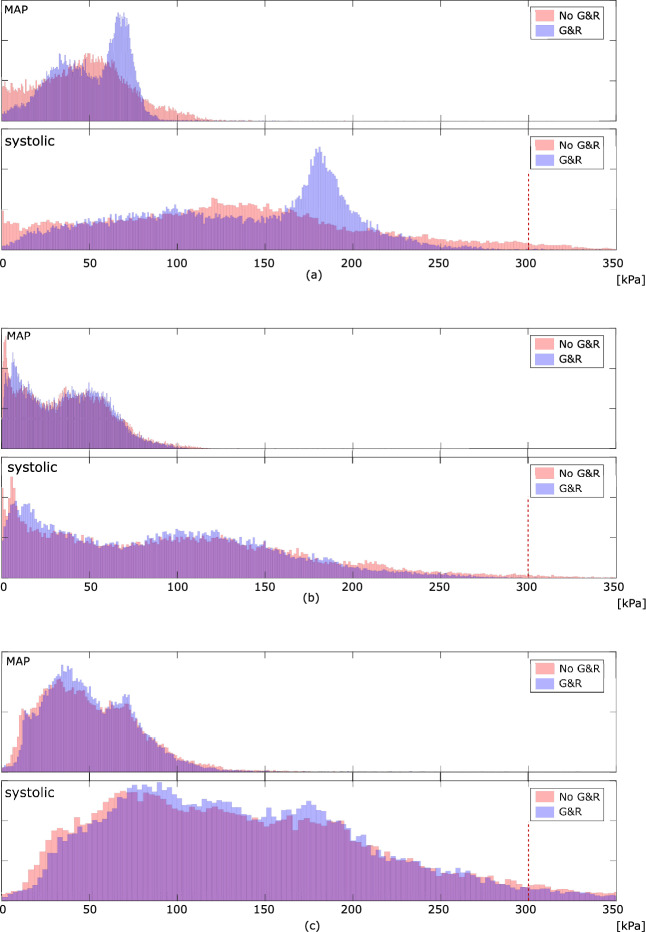
Distribution of the first principal Cauchy stress in the vessel wall at the Mean Arterial Pressure (MAP) of 75 mmHg and the systolic pressure of 180 mmHg, for Patient 2 (**a**), Patient 3 (**b**) and Patient 4 (**c**), respectively

#### Comparison Between Different Patients

In addition to the aforementioned case, we studied the implication of residual strain on wall stress predictions in three more patient-specific carotids. These cases had remarkably different levels of stenosis and wall morphologies, see Fig. 8. While growth within MATX always reduced stress peaks, this effect appeared to be negligible in vessels of either little and very large levels of stenosis, see Patients 3 and 4. However, the result is likely also influenced by the particular wall morphology. In fact, stress homogenization appears more effective for Patient 2 as for Patient 1, see Fig. 10. In contrast to Patient 1, Patient 2 exhibited a more pronounced implication of residual stresses, enabling the maintenance of a quasi-homogeneous stress distribution even at the systolic blood pressure and a significantly lower peak stress with respect to traditional computations without G&R, compare Figs. 7 and 10(top). Noticeable and similar to Patient 1, also in Patient 2, a spot of high stress remained in the fibrous cap, but here, the spot appeared close to, but not directly underneath the LRNC/IPH tissue. As expected from the very different plaque geometries and morphologies, local growth within the plaque is also very different across our cases, see Fig. 9.

## Discussion and Conclusion

We proposed an efficient simulation pipeline to compute wall stress in blood vessels upon incorporating residual strains. Homogenizing the stress in the metabolically active tissue segment (MATX) at MAP loading, determined the evolution of residual strains. We investigated atherosclerotic carotids by postprocessing image data from routine clinical examination (CT-A), and standard commercial software may be used to implement our pipeline.

The individual wall morphology played a pivotal role in how residual strains alter wall stress, and we observed also very different results depending upon the level of stenosis. Given moderate levels of stenosis, the incorporation of residual strains reduced the maximum wall stress by up to $30\%$ and resulted in a fundamentally different distribution of stress across atherosclerotic walls, as compared to a purely elastic analysis. The significance of these observations could have fundamental implications on carotid biomechanics.

Regardless residual strains levels-out tissue stress, the fibrous plaque cap may persistently be exposed to spots of high stress, one of the most interesting (and nonintuitive) findings of our study. Spots of high stress located underneath soft and thrombogenic material (LRNC/IPH) indicate risk, and carotid plaque rupture may then result in cardiovascular events, such as cerebral embolism and stroke. As tissue stress is influenced by factors, such as blood pressure, vessel geometry, tissue morphology/properties, and residual strains, the analysis of purely imaging-based information may be insufficient for robust risk prediction. The identification of high risk plaques is of paramount clinical importance, and the aforementioned limitations underline the value of a biomechanics-based carotid plaque risk assessment.

Our work is characterized by numerous limitations and a validation of our stress predictions is difficult, if not impossible. However, looking at the vessel’s pressure-free configuration, we generally observed negative and positive strains within luminal and adventitial tissue, respectively. In addition, the observed residual strain values (and opening angle) are confirmed by data reported the literature [19, 33, 34]. Regardless of being important to study, the investigation to what degree the inclusion of residual stress would improve the predictability of plaque rupture, goes well beyond the scope of the present study.

As the mechanical factors that influence vascular tissue G&R are studied incompletely, it remains unknown which parameters determine the evolution of the growth tensor ${\textbf{G}}$, and an infinite number of choices can be made. Our model was based on the first principal Cauchy stress, and it then coincides with classical 1D G&R models in the description of an infinite long thin-walled tube [5]. In addition, we applied a multiplicative kinematics-based description [25] of tissue G&R, although the tissue turn-over-based description [35] more directly describes the deposition and removal of tissue. Numerous variations of these concepts have been introduced and applied to describe G&R of vascular tissue [5]. Tissue G&R is driven by mechanical and chemical factors, and a more holistic description [36–38] as presented in this work, could result in deeper understanding and more versatile prediction of residual strains. As tissue turnover changes significantly between normal and diseased tissues, it might be important to link G&R stimuli to the underlying physiological and pathological mechanisms, respectively.

The regularization parameter *c* in the computation of the growth tensor ${\textbf{G}}$ governs the rate at which ${\textbf{G}}$ is allowed to change. Regardless the range $c \in [2,10]$ resulted in an robust algorithm for all our investigated patients, *c* is problem-depended and could require re-calibration in other cases.

The constitutive properties of the individual tissue components of the carotid vessels that have been studied in this work, were unknown. Carotid plaque tissue properties vary by orders of magnitude [39], and the morphological classification (delineation of mechanically distinct tissues) is challenging. However, promising studies have shown the possibility of detecting mechanical properties in vivo for both pathologic [40] and non-pathologic [41] vascular tissue, know-how that could be integrated into our simulation pipeline. The in vivo identification of mechanical plaque properties would most likly strengthen the robustness of the stress predictions toward more accurately identifying plaques that are vulnerably for plaque rupture.

As the primary objective of our study was to investigate the implications of residual strains, we assumed, for simplicity, isotropic tissue properties and used material parameters reported in the literature. The tissue’s non-linear stress–strain properties directly result in a highly non-linear structural problem, and our (quantitative) results are expected to be sensitive to the choice of material parameters; the study of said sensitivity was, however, beyond the scope of the present work. In addition, our calculations required the specification of algorithmic parameters, such as *c* and blood pressure increments. These are purely numerical parameters affecting the computational efficiently/robustness, but should not have implications on the final results.

Finally, the purely structural analysis of carotid vessels represents another limitations of our study. While wall shear stress from blood flow cannot alter the stress within the vessel wall, the drop of blood pressure across the stenosis in highly stenotic carotids could, however, have recognizable implications. Computational Fluid Dynamics (CFD) would allow to compute the pressure drop, where the individual inflow condition, the rheological properties of blood, and the turbulence formed within highly stenotic vessels complicate such computations.
